# Xylapeptide A, an Antibacterial Cyclopentapeptide with an Uncommon *L*-Pipecolinic Acid Moiety from the Associated Fungus *Xylaria* sp. (GDG-102)

**DOI:** 10.1038/s41598-017-07331-4

**Published:** 2017-07-31

**Authors:** Wei-Feng Xu, Xue-Mei Hou, Fei-Hua Yao, Na Zheng, Jun Li, Chang-Yun Wang, Rui-Yun Yang, Chang-Lun Shao

**Affiliations:** 1 State Key Laboratory for the Chemistry and Molecular Engineering of Medicinal Resources, College of Chemistry and Pharmaceutical Sciences, Guangxi Normal University, Guilin, 541004 The People’s Republic of China; 2 Key Laboratory of Marine Drugs, The Ministry of Education of China, School of Medicine and Pharmacy, Ocean University of China, Qingdao, 266003 The People’s Republic of China; 3 Laboratory for Marine Drugs and Bioproducts, Qingdao National Laboratory for Marine Science and Technology, Qingdao, 266200 The People’s Republic of China

## Abstract

Two new cyclopentapeptides, xylapeptide A (**1**) with an uncommon *L*-pipecolinic acid moiety, and xylapeptide B (**2**) having a common *L*-proline residue were identified from an associated fungus *Xylaria* sp. isolated from the Chinese medicinal plant *Sophora tonkinensis*. Their planar structures were elucidated by a comprehensive analysis of NMR and MS spectroscopic spectra. The absolute configurations were determined by Marfey’s method and single-crystal X-ray diffraction (Cu K*α*) analysis. Xylapeptide A (**1**) is the first example of cyclopentapeptide with *L*-Pip of terrestrial origin and showed strong antibacterial activity against *Bacillus subtilis* and *B. cereus* with MIC value of 12.5 *μ*g/mL.

## Introduction

New antibiotics are in need of development against the resistant microorganisms in controlling bacterial infections^1, 2^. Endophytes are increasingly attracting attention as sources of potentially valuable compounds^3^. The secondary metabolites produced by endophytes have received enduring development for chemical diversity and antimicrobial activities^2, 4^. Peptides are investigated intensively because they are target specific with higher degree of interactions^5^. In recent years, a quite number of peptides isolated from endophytes have been reported. It is noteworthy that peptides with good antimicrobial activities were isolated mainly from *Streptomyces* genus, such as kakadumycin A^6^ showed potent antibacterial activity against *Staphylococcus aureus*, *S. simulans*, *S. pneumonia*, and *Shigella dysenteriae*; munumbicin C^7^ displayed strong activity against vancomycin-sensitive *S. aureus*; actinomycin D^8^ exhibited strong activity against methicillin-resistant *S. aureus* and multidrug-resistant *Mycobacterium tuberculosis*, and munumbicin E-4^8^ demonstrated strong activity against *S. aureus*. However, few peptides isolated from fungi exhibited good antimicrobial activities, the example was the cyclo-(Pro-Thr) and cyclo-(Pro-Tyr)^9^ (produced by a *Penicillium* sp. isolated the mangrove *Acrostichum aureurm*) showed a strong antibacterial activity against *S. aureus* and antifungal activity against *Candida albicans*.

**Figure 1:**
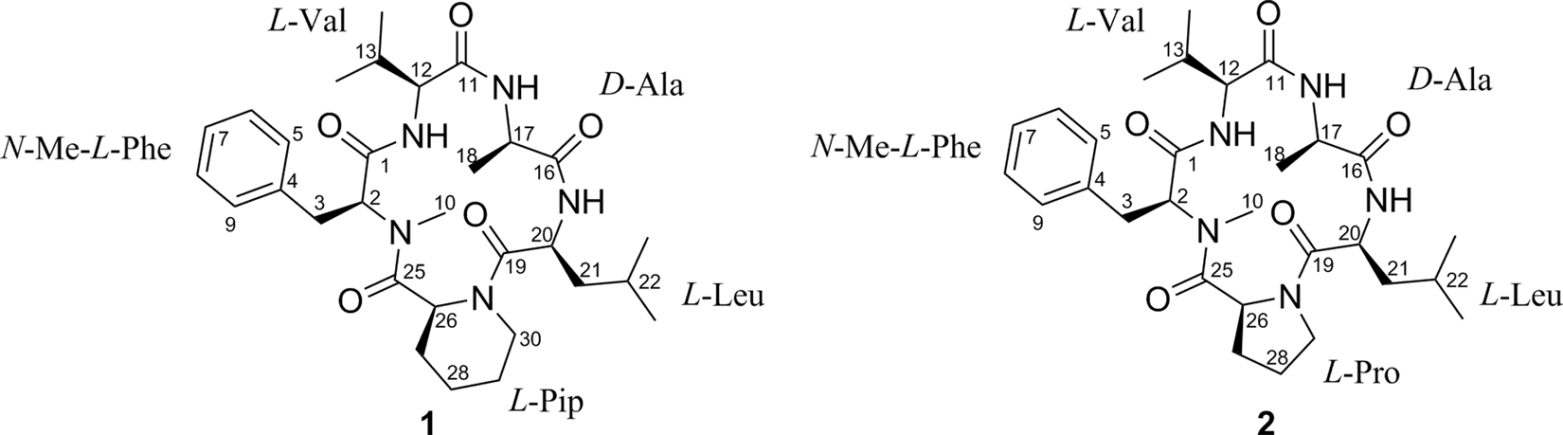
Chemical structures of xylapeptides A (**1**) and B (**2**).

*Sophora tonkinensis* is an important traditional Chinese medicinal plant used widely to treat acute pharyngolaryngeal infections and sore throats^10, 11^. In the course of our studies to investigate antimicrobial constituents from associated fungi, the fungus *Xylaria* sp. (GDG-102) was isolated from *S. tonkinensis*. The crude extract of the fermentation broth exhibited antibacterial activity. Preliminary UPLC-MS and ^1^H NMR analysis showed that this fungus produced relatively polar peptide compounds (Figure S1). Chemical investigation led to the identification of two new cyclopentapeptides, xylapeptides A (**1**) and B (**2**) (Fig. 1). Xylapeptide A is the first cyclopentapeptide with *L*-Pip of terrestrial origin, and exhibited strong antibacterial activity against *Bacillus subtilis* and *B. cereus*. Xylapeptide B showed strong antibacterial activity against *B. subtilis*, *B. cereus*, *B. megaterium*, *Micrococcus luteus*, *S. aureus*, *Shigella castellani*, as well as strong antifungal activity against *C. albicans*. Herein, we report the structure determination and biological activities of the two cyclic peptides.

## Results and Discussion

**Figure 2:**
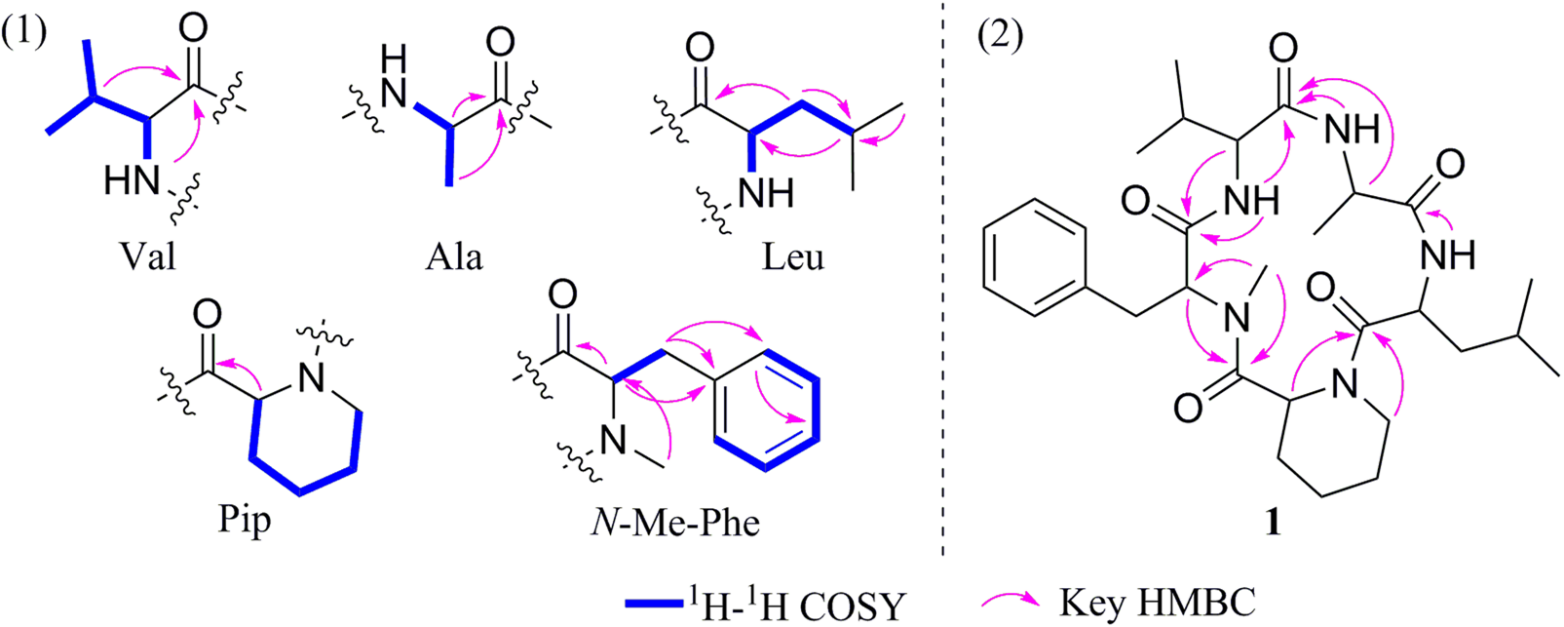
(**1**) the subunits of 1 derived from 2D NMR data and (**2**) the assemble of the subunits by key HMBC correlations.

Xylapeptide A (**1**) was obtained as a colorless crystal. Its HRESIMS gave a [M + H]^+^ at *m*/*z* 556.3505, indicating a molecular formula of C_30_H_45_N_5_O_5_ and requiring 11 degrees of unsaturation. The ^1^H NMR spectrum (Table 1) suggested that it has three amide (NH) protons at *δ*_H_ 7.45, 7.11, and 6.13, one *N*-methyl group at *δ*_H_ 2.97, and four *α*-amino protons at *δ*_H_ 4.74, 4.61, 4.34, and 4.14. It also showed a single mono-substituted benzene at *δ*_H_ 7.31, 7.26 and 7.17. The ^13^C NMR spectrum indicated that it has five amide type carbonyls at *δ*_C_ 172.8, 172.6, 171.8 (2 C) and 170.2, six nitrogenated sp^3^ carbon resonance at *δ*_C_ 64.2, 59.0, 57.0, 48.1, 47.5 and 47.4. These above NMR features accounted for 9 of the 11 unsaturations. Further analysis of 2D NMR spectrum allowed five subunits to be established, an *N*-Me-phenylalanine (*N*-Me-Phe), a valine (Val), an alanine (Ala), a leucine (Leu), and a pipecolinic acid (Pip) (Fig. 2). Compound **1** was concluded to be a cyclopeptide on the basis of these spectral characteristics.

**Table 1:** NMR data of xylapeptide A (1)^a^.

Amino acid	Position	*δ*_C_	*δ*_H_ (*J* in Hz)	COSY	HMBC
*N*-Me-Phe	1	170.2			
	2	64.2	4.34, d (10.7)	H-3	C-1/3/10/25
	3	34.3	2.82, dd (13.5, 10.7) 3.62, d (13.5)	H-2	C-2/4/5/9
	4	138.5			
	5/9	129.5	7.17, d (7.2)		
	6/8	129.1	7.31, t (7.2)		C-4
	7	127.2	7.26, m		
	10	31.6	2.97, s		
Val	11	171.8			
	12	59.0	4.14, t (9.0)	H-13,NH	C-1/11/13/14
	13	29.0	2.23, m	H-2	
	14	18.7	0.96, d (6.8)		
	15	19.7	0.98, d (6.8)		
	NH		7.45, d (9.0)	H-12	C-1/12
Ala	16	172.8			
	17	47.5	4.61, m	H-18, NH	C-18
	18	14.2	1.32, d (6.8)	H-17	C-16/17
	NH		6.13,d (8.2)	H-17	C-11
Leu	19	172.6			
	20	48.1	4.74, t (8.4)	NH	C-19/21
	21	41.1	1.25, m 1.55, m		
	22	24.8	1.59, m		
	23	21.5	0.88, d (6.3)		C-21/22
	24	23.3	0.89, d (6.3)		
	NH		7.11, d (8.4)	H-20	C-16
Pip	25	171.8			
	26	57.0	2.44, d (10.4)	H-27	C-25/27
	27	28.5	0.70, m 1.40, m	H-28	
	28	23.2	0.79, m 1.57, m	H-29	
	29	25.2	1.31, m 1.55, m	H-30	
	30	47.4	2.73, dd (13.3, 9.0) 3.88, d (13.3)	H-29	C-19

^a^Measured at 500 MHz for ^1^H NMR and 125 MHz for ^13^C NMR in CDCl_3_.

**Figure 3:**
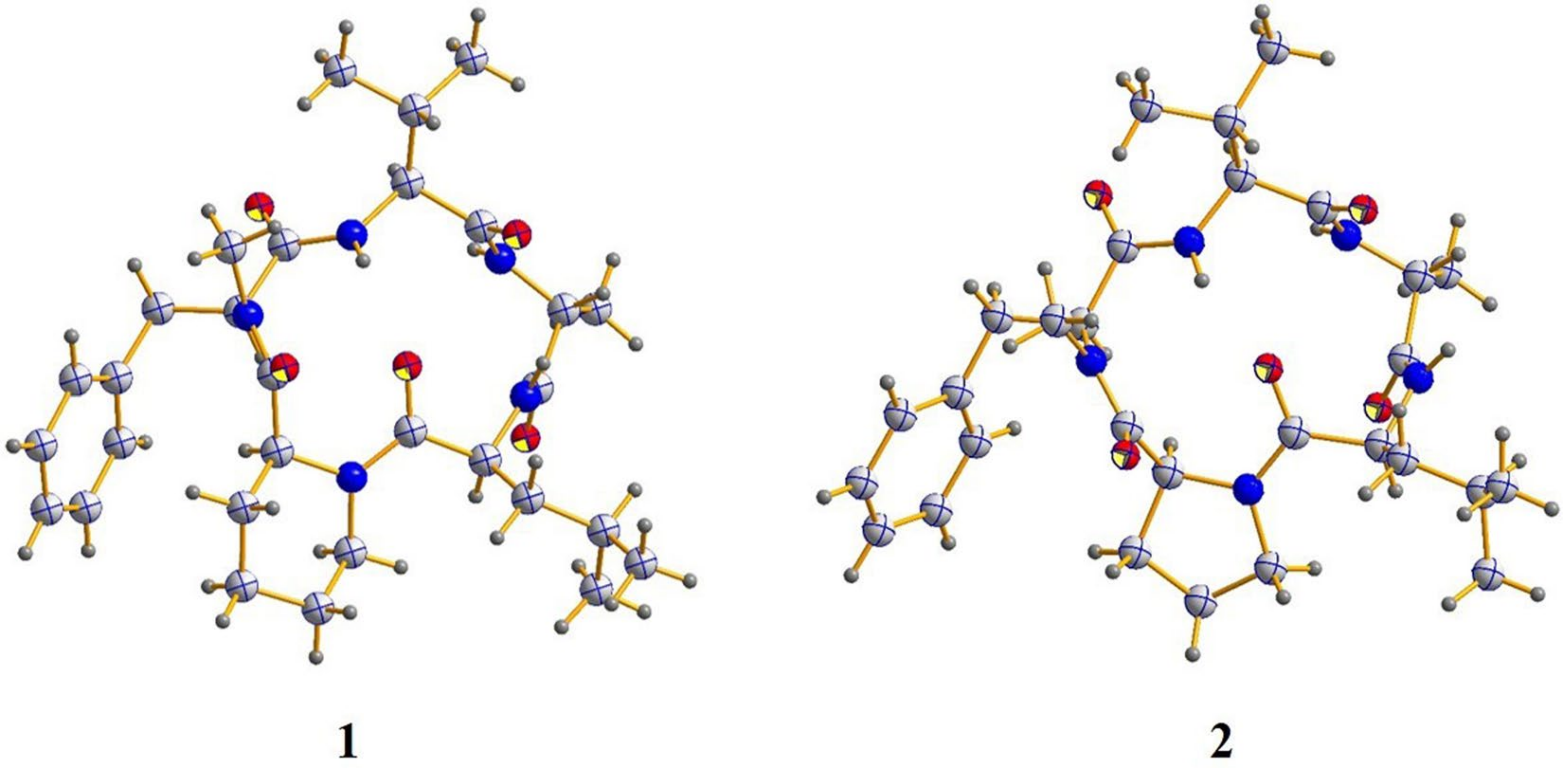
X-ray crystallographic analysis of (**1** and **2**).

The sequence of residues in **1** was elucidated on the basis of HMBC correlations (Fig. 2). The HMBC correlations from Pip-H-6 to Leu-CO, Leu-NH to Ala-CO, Ala-NH to Val-CO, Val-NH to *N*-Me-Phe-CO, and *N*-Me-Phe-*α*-H to Pip-CO allowed the definition of the residue sequence of **1** as *cyclo*-(CO-Pip → Leu → Ala → Val → *N*-Me-Phe-N). Furthermore, the ESI MS^2^ (Fig. S16) fragment ions corresponding to neutral losses of [Pip], [Pip−Leu], [Pip−Leu−Ala], and [Pip−Leu−Ala−Val] were also observed which confirmed the cyclic structure for **1**. The configurations of the amino acid residues of **1** was determined by acid hydrolysis, derivatization with *N* ^α^-(2,4-dinitro-5-fluorophenyl)-*L*-alalinamide (*L*-FDAA, the advanced Marfey’s method^12^), and UPLC-MS analysis of the derivatives with comparison to the standards (Figure S17). Retention times (min) of the standard amino acid derivatives were as follows: *L*-Ala, *D*-Ala, *L*-Val *D*-Val, *L*-Leu, *D*-Leu, *N*-Me-*L*-Phe and *N*-Me-*D*-Phe were 5.817, 6.811, 7.692, 8.977, 8.996, 10.212, 9.281, and 9.736 min, respectively. Retention times of the derivatives of the acid hydrolysate of **1** were 6.793, 7.676, 8.975, and 9.263 min. It indicated that Leu, Val, and *N*-Me-Phe had the *L*-configuration, while Ala was *D*-configured. Finally, the absolute configuration of **1** was confirmed unambiguously by single-crystal X-ray analysis which indicated an *L*-configuration of Pip (Fig. 3).

**Figure 4:**
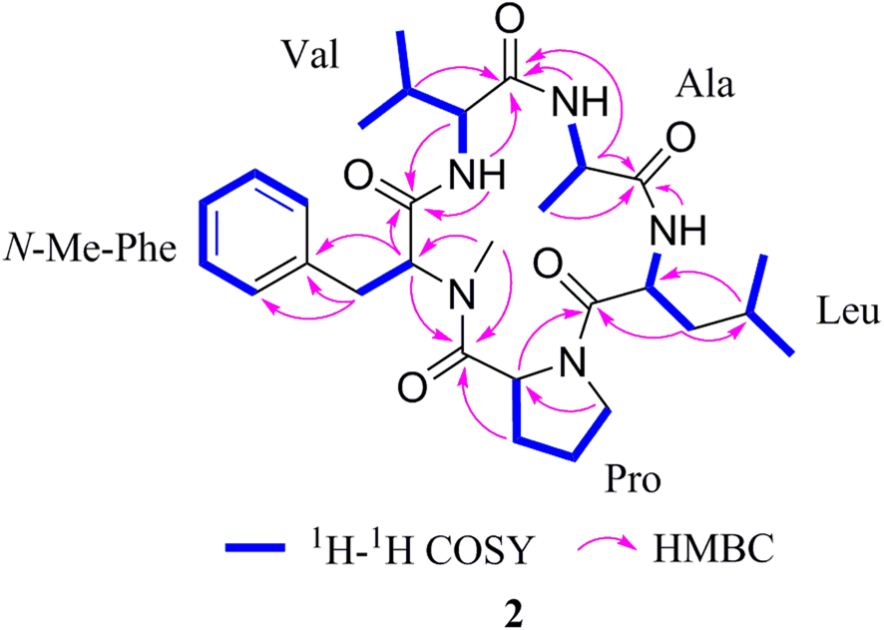
^1^H-^1^H COSY and HMBC correlations of 2.

Xylapeptide B (**2**) was also obtained as a colorless crystal. Its molecular formula C_29_H_43_N_5_O_5_ (eleven degrees of unsaturation) was determined on the basis of HRESIMS data. The ^1^H and ^13^C NMR spectrum (Table 2) indicated that **2** was very similar to **1** with the additional high-field resonances (*δ*_H_ 0.79/1.57, *δ*_C_ 23.2 (CH_2_)) in **1** were absent. Examination of the 2D NMR spectra revealed that **2** shared the same *N*-Me-Phe, Val, Ala, and Leu units with **1**, the only difference is that the Pip in **1** was replaced by a proline (Pro) subunit in **2**. The sequence of the amino acids of **2** was also assigned by HMBC correlations (Fig. 4) and fully supported by the ESI MS^2^ experiment results (Fig. S16) which revealed the same linkage with **1**. The configurations of the amino acid residues were also determined by Marfey’s method. The retention times (min) of the standard *L*-Pro and *D*-Pro were 6.262 and 6.689 min, respectively. The retention times for the acid hydrolysate of **2** were 6.285, 6.818, 7.707, 9.004, and 9.318 min, respectively, which correspond to *L*-Pro, *L*-Leu, *L*-Val, *N*-Me-*L*-Phe, and *D*-Ala (Figure S17). Besides, by slow crystallization from CH_3_OH, single crystals of **2** suitable for X-ray diffraction analysis using Cu K*α* radiation was also obtained, allowing the complete structure to be established unambiguously (Fig. 3).

**Table 2:** NMR data of xylapeptide B (2)^a^.

Amino acid	Position	*δ*_C_	*δ*_H_ (*J* in Hz)	COSY	HMBC
*N*-Me-Phe	1	170.1			
	2	64.9	4.93, dd (12.1, 2.6)	H-3	C-1/3/4/10
	3	34.1	2.83, dd (14.2, 12.1) 3.60, dd (14.2, 2.6)	H-2	C-2/4/5/9
	4	138.4			
	5/9	129.4	7.10, d (7.2)	H-6/8	
	6/8	128.9	7.26, t (7.2)	H-5/9	
	7	126.8	7.20, t (7.2)		
	10	30.8	2.93, s		C-2/25
Val	11	171.1			
	12	60.0	4.14, t (8.2)	H-13, NH	C-11/13/14
	13	30.2	2.23, qd (13.5, 6.8)	H-12/14	C-11/12/14
	14	18.6	0.96, d (6.8)	H-13	C-12/13
	15	19.8	0.95, d (6.8)	H-13	C-12/13
	NH		8.23, d (8.2)	H-12	C-1/11/12
Ala	16	174.6			
	17	48.3	4.56, dq (8.5, 6.9)	H-18, NH	C-11/16/18
	18	14.8	1.29, d (6.9)	H-17	C-16/17
	NH		6.14, d (8.5)	H-17	C-11/17/18
Leu	19	170.6			
	20	52.1	4.32, td (10.2, 3.9)	H-21, NH	C-19/21/23
	21	38.4	1.41, m	H-2	C-19/20/22/23
	22	25.2	1.72, m	H-5	C-20/21/23
	23	21.1	0.88, d (6.5)	H-4	C-20/22
	24	23.3	0.90, d (6.5)	H-4	C-20/22
	NH		6.46, d (10.2)	H-2	C-16/21
Pro	25	172.6			
	26	56.4	3.88, dd (9.2, 6.4)	H-27	C-19/25/27
	27	28.3	1.45, m 0.52, dt (6.4, 3.9)	H-26/28	C-25/28/29
	28	25.7	1.49, m 1.89, m	H-29	C-26/27
	29	46.2	3.33, ddd (14.0, 9.7, 6.7) 3.48, dd (14.0, 6.7)	H-28	C’26/27/28

^a^Measured at 400 MHz for ^1^H NMR and 100 MHz for ^13^C NMR in CDCl_3_.

Naturally occurring cyclic peptides with nonproteinogenic amino acids mainly contained pipecolinic acid (Pip) and anthranilic acid (Ant)^13^. Up to now, nearly twenty cyclic peptides with Pip residues isolated from nature sources have been reported which occupied a very small proportion of the identified peptide. Most of the cyclic peptides with Pip residues reported so far were of a marine origin^14, 15^ such as cyclopentadepsipeptides JBIR-113, 114 and 115 (fungus *Penicillium* sp^16^.), apratoxin H (cyanobacterium *Moorea producens* ^17^), petrosifungins A and B (fungus *Penicillium brevicompactum* ^18^), cyclohexapeptide similanamide (fungus *Aspergillus similanensis* ^19^), cyclohexadepsipeptides somamides A and B (cyanobacteria *Lyngbya majuscule* and *Schizothrix* sp^20^.), symplostatins (cyanobacterium *Symploca hydnoides* ^21^), tasipeptins A and B (cyanobacterium *Symploca* sp^22^.), kurahamide (cyanobacterium *Moorea* sp^23^.), and dolastatins (sea hare *Dolabella auricularia* ^24^), cycloheptadepsipeptides neamphamides B−D (sponge *Neamphius huxleyi* ^25^), cyclooctadepsipeptide homophymine A (sponge *Homophymia* sp^26^.), and pipecolidepsins A and B (sponge *Homophymia lamellose* ^27^). Cyclic peptides with Pip from terrestrial origins are rare including cyclohexapeptides PF1171 A−G (unknown ascomycete OK-128^13^), and cyclodecadepsipeptides, clavariopsins A and B (aquatic hyphomycetes *Clavariopsis aquatic* ^28^). Up to now, the Pip in all the peptides is *L*-configuration. Xylapeptide A (**1**) is the first cyclopentapeptide from a terrestrial origin with *L*-Pip.

The transformation of Lys to Pip in plants and fungus has been studied by radioactive labeling experiment. In the Ca-deficient wheat plants, the ^14^C-*L*-Lys was transported rapidly in to the shoots and then degraded to Pip in many pathways^29, 30^. In the *Rhizoctonia leguminicola* fungus, the *L*-[U-^14^C]-Lys is used to form *L*-Pip^31, 32^. Although Gatto and co-workers reported the first *in vitro* characterization of a lysine cyclodeaminase (RapL) which utilized the NAD^+^ as a cofactor in a catalytic manner in *Streptomyces hygroscopicus* to catalyst lysine into Pip^33^, the genes controlling the biosynthesis of Lys to Pip have not been described yet. It is suggested that the Pip in xylapeptide A (**1**) may also be converted from lysine, and a radioactive labeling experiment is needed.

Xylapeptides A (**1**) and B (**2**) were evaluated for their antibacterial activity. Compound **1** showed strong and selective antibacterial activity against *Bacillus subtilis* and *B. cereus* with MIC value of 12.5 *μ*g/mL. Compound **2** exhibited strong and broad antibacterial spectrum against *B. subtilis*, *B. cereus*, *B. megaterium*, *Micrococcus luteus*, *Staphylococcus aureus*, and *Shigella castellani* with MIC values of 12.5, 6.25, 6.25, 12.5, 12.5, and 12.5 *μ*g/mL, respectively. Compound **2** also showed a strong antifungal activity against *Canidia albicans* with MIC value of 12.5 *μ*g/mL. This suggested that the *L*-Pip residue and *L*-Pro residue play different roles in the antimicrobial activity. The cytotoxic, *α*-glucosidase inhibitory, and antiviral activities of **1** and **2** were also evaluated. However, they were all inactive.

In summary, two new cyclopentapeptides, xylapeptides A (**1**) and B (**2**), were isolated from a *Xylaria* sp. fungus cultured from the Chinese medicinal plant *S. tonkinensis*. Xylapeptide A (**1**) contains a non-proteinogenic amino acid, *L*-pipecolinic acid (*L*-Pip), which is the first example of cyclopentapeptide from a terrestrial origin with *L*-Pip. Compound **1** displayed a strong antibacterial activity against *Bacillus subtilis* and *B. cereus*. Xylapeptide B (**2**) has a broader antibacterial spectrum as well as antifungal activity.

## Methods

### General experimental procedures

Optical rotations were measured on a JASCO P-1020 digital polarimeter. UV spectra were determined on a Shimadzu double-beam 210 A spectrometer. IR spectra were taken on a Bruker EQUINOX 55 spectrometer using KBr pellets. 1D and 2D NMR spectra were obtained on an Agilent DD2 400 MHz NMR spectrometer or a DRX-500 spectrometer, respectively, with TMS as the internal standard. ESIMS and HRESIMS spectra were obtained from a Micromass Q-TOF spectrometer and a Thermo Scientific LTQ Orbitrap XL spectrometer. Semi-preparative HPLC was performed on a Waters 1525 system using a C18 (Kromasil, 5 *μ*m, 10 × 250 mm) column coupled with a Waters 2996 photodiode array detector. UPLC MS was performed on Waters UPLC^®^ system using a C18 column [ACQUITY UPLC^®^ BEH C18, 2.1 × 50 mm, 1.7 μm; 0.5 mL/min] and ACQUITY QDa ESIMS scan from 150 to 1000 Da. Silica gel (Qing Dao Hai Yang Chemical Group Co.; 200–300 mesh), and octadecylsilyl silica gel (Unicorn; 45–60 μm), were used for column chromatography (CC). Precoated silica gel plates (Yan Tai Zi Fu Chemical Group Co.; G60, F-254) were used for thin-layer chromatography.

### Isolation and identification of the fungal material

The fungus *Xylaria* sp. (strain # GDG-102) was isolated from the Chinese medicinal plant *S. tonkinensis* collected in Hechi, Guangxi Province, in October, 2014. The fungus *Xylaria* sp. (strain # GDG-102) was isolated from fresh healthy leaves of Chinese medicinal plant *S. tonkinensis* collected in Hechi, Guangxi Province, in October, 2014. The leaves were designed to undergo a process described as surface sterilization^34^. The surface-sterilized leaves were aseptically sectioned into small pieces and plated onto the PDA plates containing an antibiotic to suppress bacterial growth (composition of isolation medium: potatoes 200 g/L, glucose 20 g/L, agar 15 g/L and ampicillin sodium 0.2 g/L in water). After incubation at 27 °C, the fungal strain under investigation was isolated from the growing cultures by repeated reinoculation on PDA plates. The fungus was identified as *Xylaria* sp. (GenBank accession number KU645984) using a molecular biological protocol by DNA amplification and sequencing of the ITS region as described in ref. 34.

### Fermentation, extraction, and isolation

The fungal strain was cultivated in 20 L of liquid medium (composition of medium: 200 g/L cooked and sliced potatoes, 20 g/L glucose in water, in 1 L Erlenmeyer flasks each containing 500 mL of culture broth) at room temperature without shaking for 7 weeks. Then the culture was filtered to separate the culture broth from the mycelia. The culture broth was extracted with an equal volume of ethyl acetate (EtOAc) and the fungal mycelia were extracted with CH_2_Cl_2_-CH_3_OH (v:v, 1:1) for three times, respectively. The mycelia extraction were concentrated to about 0.3 L and extracted with EtOAc. The EtOAc layer was evaporated to dryness under reduced pressure to give broth extract (4.5 g) and mycelia extract (4.8 g), respectively. The broth extract was subjected to silica gel CC (petroleum ether (PE)−EtOAc, v-v, gradient) to afford five fractions (Fr.1–Fr.5). Fr.4 was repeated purified by ODS CC (CH_3_OH−H_2_O) and then recrystallized in MeOH to obtain **2** (20 mg). In search for similar compounds, the mycelia extraction was subjected to silica gel CC (PE−EtOAc, v:v, gradient) to afford six fractions (Fr.1–Fr.6). Under the guidance of ^1^H NMR, Fr.4 was chromatographed on ODS with CH_3_OH−H_2_O, and then purified by semi-preparative HPLC (CH_3_OH-H_2_O, v:v, 83:17) to yield **1** (10 mg).

*Xylapeptide A (* ***1*** *)*. colorless crystals; ${[\alpha ]}_{{\rm{D}}}^{20}$ −15.6 (*c* 0.1, CH_3_OH); UV (CH_3_OH) *λ*_max_ (log *ε*) 205 (4.44) nm; IR (KBr) *ν*_max_ 3446, 1683, 1521 cm^−1^; ^1^H and ^13^C NMR data, see Table 1. ESI MS^2^ (fragmentation of *m*/*z* 556.4 [M + H]^+^) *m*/*z* 445.2 [M − Pip + H]^+^, 332.2 [M − Pip − Leu + H]^+^, 261.1 [M − Pip − Leu − Ala + H]^+^, 162.1 [M − Pip − Leu − Ala − Val + H]^+^; HRESIMS *m*/*z* 556.3505 [M + H]^+^ (calcd for C_30_H_46_N_5_O_5_, 556.3493).

*Xylapeptide B (* ***2*** *)*. colorless crystals; ${[\alpha ]}_{{\rm{D}}}^{20}$ − 85.7 (*c* 0.2, CH_3_OH); UV (CH_3_OH) *λ*_max_ (log *ε*) 206 (4.47) nm; IR (KBr) *ν*_max_ 3434, 2959, 1648, 1523, 1454 cm^−1^; ^1^H and ^13^C NMR data, see Table 2; ESI MS^2^ (fragmentation of *m*/*z* 542.3 [M + H]^+^) *m*/*z* 443.2 [M − Val + H]^+^, 372.2 [M − Val − Ala + H]^+^, 259.1 [M − Val − Ala − Leu + H]^+^, 162.0 [M − Val − Ala − Leu − Pro + H]^+^; HRESIMS *m*/*z* 542.3345 [M + H]^+^ (calcd for C_29_H_44_N_5_O_5_, 542.3337).

### X-ray crystallographic analysis of compounds 1 and 2

Upon crystallization from CH_3_OH-H_2_O, the regular needle crystals of **1** and **2** were obtained. The single-crystal X-ray diffraction data were collected at 293(2) K for **1** and 296.15 K for **2** on a Bruker APEX-II CCD diffractometer with Cu K*α* radiation (*λ* = 1.54178 Å). Crystallographic data for **1** and **2** have been deposited with the Cambridge Crystallographic Data Centre. Copies of the data can be obtained, free of charge, on application to the Director, CCDC, 12 Union Road, Cambridge CB2 1EZ, UK (fax: + 44-(0)1223-336033, or e-mail: deposit@ccdc.cam.ac.uk).

*Crystal data for* **1**: C_30_H_45_N_5_O_5_∙2(H_2_O) M*r* = 591.74, monoclinic, space group P2(1) with *a* = 13.3173 (2) Å, *b* = 6.90755 (8) Å, *c* = 17.8276 (3) Å, *α* = 90.00°, *β* = 93.7299 (14)°, *γ* = 90.00°, *V* = 1636.50 (4) Å^3^, *Z* = 2, *D*_x_ = 1.201 mg/m^3^, *μ* (Cu K*α*) = 1.54178 mm^−1^, and *F*(000) = 640. Crystal dimensions: 0.40 × 0.28 × 0.10 mm^3^. Independent reflections: 20526, the final *R*_1_ value was 0.0337, *wR*_2_ = 0.0824 (*I* > 2*σ*(*I*)), Flack parameter = 0.04(15). CCDC number: 1501650.

*Crystal data for* **2**: C_29_H_43_N_5_O_5_∙H_2_O∙CH_3_OH, M*r* = 591.74, monoclinic, space group P2(1) with *a* = 9.9670 (5) Å, *b* = 12.5923(7) Å, *c* = 26.2574 (14) Å, *α* = 90.00°, *β* = 90.00°, *γ* = 90.00°, *V* = 3295.5(3) Å^3^, *Z* = 4, *D*_x_ = 1.193 mg/m^3^, *μ* (Cu K*α*) = 1.54184 mm^−1^, and *F*(000) = 1280. Crystal dimensions: 0.30 × 0.15 × 0.08 mm^3^. Independent reflections: 20526, The final *R*_1_ value was 0.0756, *wR*_2_ = 0.1010 (*I* > 2*σ*(*I*)), Flack parameter = −0.1(2). CCDC number: 1502010.

### Absolute configuration of xylapeptides A (1) and B (2)

The amino acid standards (*L*- and *D*/*L*-configurations) relevant to **1** and **2** were obtained commercially and 0.5 mg was dissolved in 20 *μ*L H_2_O. Each standard was then derivatized for Marfey’s analysis by adding 1 M NaHCO_3_ (10 *μ*L) and *L*-FDAA (1% w/v in acetone, 50 *μ*L). The mixture was heated at 45 °C for 1 h with continuous stirring, then neutralized with 1 M HCl after cooling at room temperature. The derivatives were then dried and diluted with CH_3_OH and analyzed by UPLC MS (using a C_18_ column [ACQUITY UPLC^®^ BEH C18, 2.1 × 50 mm, 1.7 *μ*m; 0.5 mL/min, UV detection at 340 nm; ACQUITY QDa ESIMS scan from 150 to 1000 Da; linear gradient: (A) CH_3_CN and (B) H_2_O with 0.1% HCOOH 5–50% A (0–13 min), 50–100% A (13–15 min), 100% A (15–17 min), 100–5% A (17–18 min), 5% A (18–20 min). Retention times (min) of the standard amino acid derivatives of *L*-Ala, *D*-Ala, *L*-Pro, *D*-Pro, *L*-Val *D*-Val, *L*-Leu, *D*-Leu, *N*-Me-*L*-Phe and *N*-Me-*D*-Phe were 5.817, 6.811, 6.262, 6.689, 7.692, 8.977, 8.996, 10.212, 9.281, and 9.736 min, respectively.

Xylapeptide A (or xylapeptide B, 0.5 mg) was hydrolyzed with 6 M HCl (2.0 mL) at 110 °C for 15 h. The solution was evaporated to dryness, and derivatized for Marfey’s analysis in a similar manner to the derivatized standard amino acids. The derivatives of the acid hydrolysate of **1** were analyzed by UPLC-MS with the retention times as follows: 6.793 (*D*-Ala), 7.676 (*L*-Val), 8.975 (*L*-Leu), and 9.263 (*N*-Me-*L*-Phe) min. The retention times of that in **2** were: 6.285 (*L*-Pro), 6.818 (*D*-Ala), 7.707 (*L*-Val), 9.004 (*L*-Leu), and 9.318 (*L*-*N*-Me-Phe) min.

### Biological assays

The antibacterial activity against *B. subtilis*, *B. cereus*, *B. megaterium*, *Micrococcus luteus*, *Staphylococcus aureus*, and *Shigella castellani*, and antifungal activity against *C. albicans* were evaluated by using 96-well microtitre plates^35^, with ampicillin as positive control. The cytotoxic activity against human colon carcinoma (HCT-116), human cervical cancer (HeLa), non-small cell lung carcinoma (A549), human breast cancer (MCF-7), human pancreatic carcinoma (BXPC-3), and chronic myelocytic leukemia (K562) cell lines was evaluated by the SRB method^36^ and MTT method^37^, with adriamycin as a positive control. The antiviral activity against human cytomegalovirus (HCMV), and herpes simplex virus type 1 (HSV-1) virus was evaluated by the cytopathic effect inhibition assay^38^, with cidofovir and acyclovir as positive control. The *α*-glucosidase inhibitory activity was evaluated by Yilmazer-Musa’s method^39^, with acarbose as a positive control.

## Electronic supplementary material

Supplementary Information
