# Supplementary material for: Xylapeptide A, an Antibacterial Cyclopentapeptide with an Uncommon L-Pipecolinic Acid Moiety from the Associated Fungus Xylaria sp. (GDG-102)
Source: Sci Rep. 2017 Jul 31;7:6937. doi: 10.1038/s41598-017-07331-4 (PMC5537251; doi:10.1038/s41598-017-07331-4)
Supplement: Supplementary file 1 — Supplementary Information [file 41598_2017_7331_MOESM1_ESM.pdf]

Xylapeptide A, an Antibacterial Cyclopentapeptide with an Uncommon *L*-Pipecolinic Acid Moiety from the Associated Fungus *Xylaria* sp. (GDG-102)

Wei-Feng Xu, [†,‡,¶] Xue-Mei Hou, [‡,§,¶] Fei-Hua Yao,[†] Na Zheng,[†] Jun Li,[†] Chang-Yun Wang, [‡,§] Rui-Yun Yang[*,†] and Chang-Lun Shao[*,‡,§]

[†]State Key Laboratory for the Chemistry and Molecular Engineering of Medicinal Resources, College of Chemistry and Pharmaceutical Sciences, Guangxi Normal University, Guilin 541004, The People's Republic of China

[‡]Key Laboratory of Marine Drugs, The Ministry of Education of China, School of Medicine and Pharmacy, Ocean University of China, Qingdao 266003, The People's Republic of China

[§]Laboratory for Marine Drugs and Bioproducts, Qingdao National Laboratory for Marine Science and Technology, Qingdao 266200, The People's Republic of China

¶ These authors contributed equally to this work

* To whom correspondence should be addressed. Tel/Fax: +86-773-2120-958 (R.-Y.Y.); +86-532-8203-1503 (C.-L.S.). E-mails: yang_rui_yun@163.com (R.-Y.Y.); shaochanglun@163.com (C.-L.S.).

List of Supporting Information

Figure S1. ^{1}H NMR spectra of the organic extracts, and xylapeptides A and B in $CDCl_3$.

Figure S2. ^{1}H NMR (500 MHz, $CDCl_3$) spectrum of compound **1**.

Figure S3. ^{13}C NMR (125 MHz, $CDCl_3$) spectrum of compound **1**.

Figure S4. ^{1}H-^{1}H COSY ($CDCl_3$) spectrum of compound **1**.

Figure S5. HSQC ($CDCl_3$) spectrum of compound **1**.

Figure S6. HMBC ($CDCl_3$) spectrum of compound **1**.

Figure S7. HRESIMS spectrum of compound **1**.

Figure S8. ESI MS^2 spectrum of compound **1**.

Figure S9. ^{1}H NMR (400 MHz, $CDCl_3$) spectrum of compound **2**.

Figure S10. ^{13}C NMR (100 MHz, $CDCl_3$) spectrum of compound **2**.

Figure S11. ^{1}H-^{1}H COSY ($CDCl_3$) spectrum of compound **2**.

Figure S12. HSQC ($CDCl_3$) spectrum of compound **2**.

Figure S13. HMBC ($CDCl_3$) spectrum of compound **2**.

Figure S14. HRESIMS spectrum of compound **2**.

Figure S15. ESI MS^2 spectrum of compound **2**.

Figure S16. Assignments of ESI MS^2 fragmentation data for **1** and **2**.

Figure S17. Marfey's analysis of the amino acids in **1** and **2** on UPLC-MS.

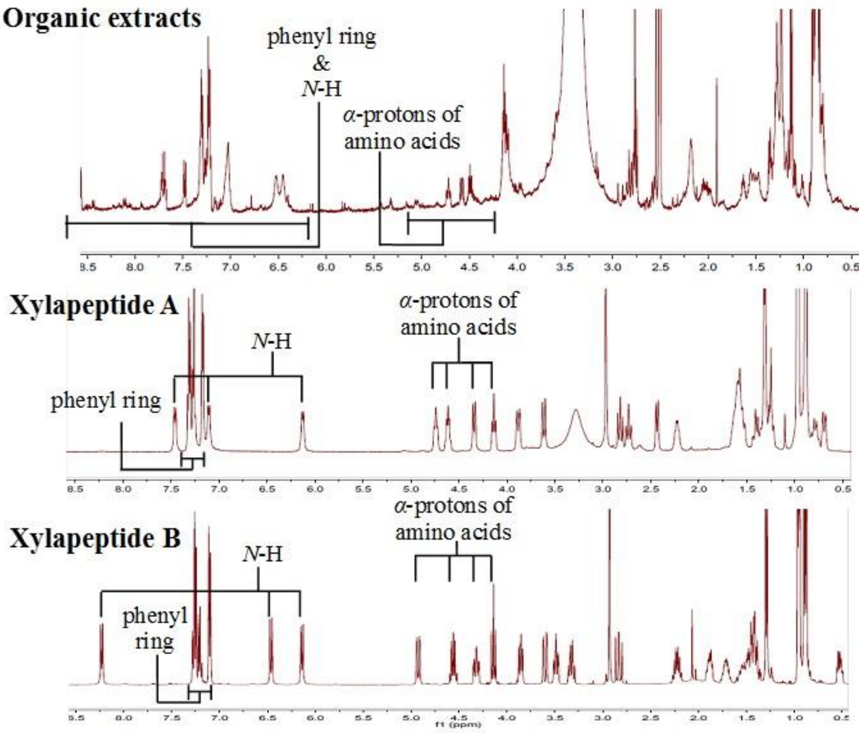

Figure S1. ^{1}H NMR spectra of the organic extracts, and xylapeptides A and B in $CDCl_3$.

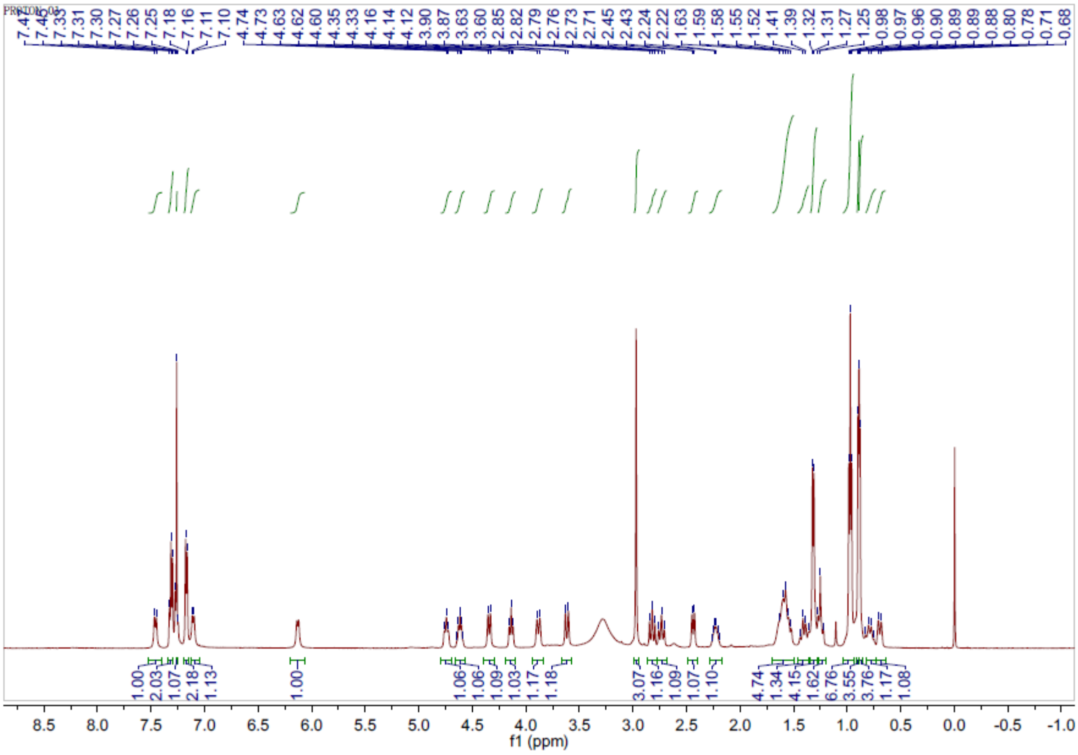

Figure S2. ^{1}H NMR (500 MHz, $CDCl_3$) spectrum of compound **1**.

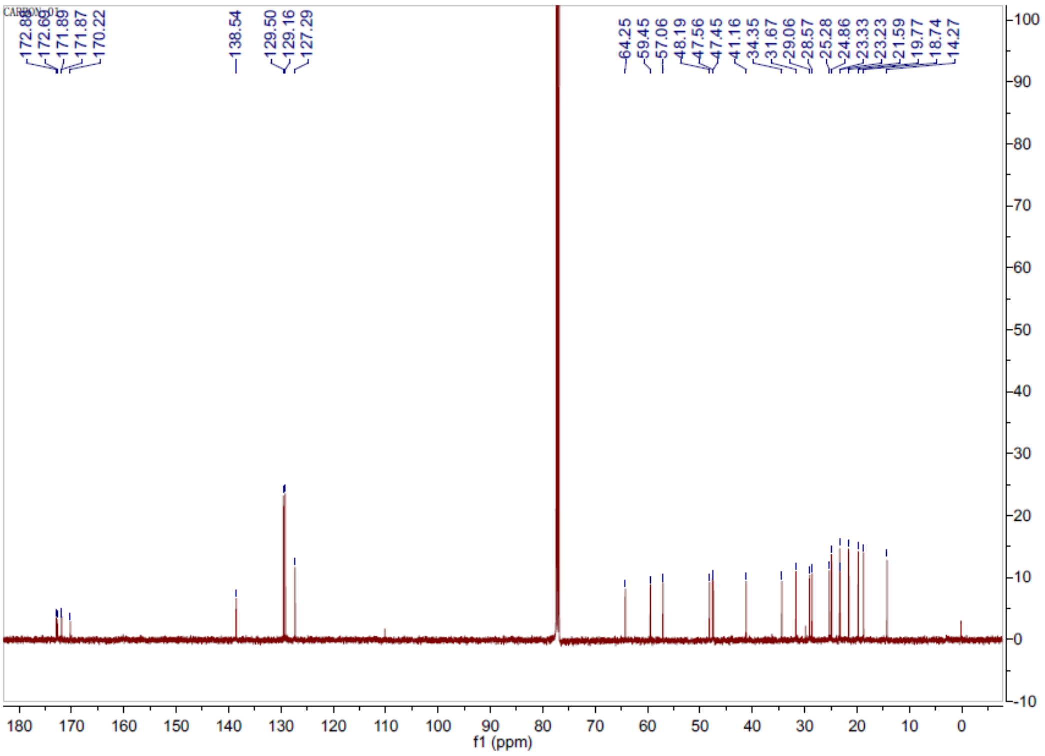

Figure S3. ^{13}C NMR (125 MHz, $CDCl_3$) spectrum of compound **1**.

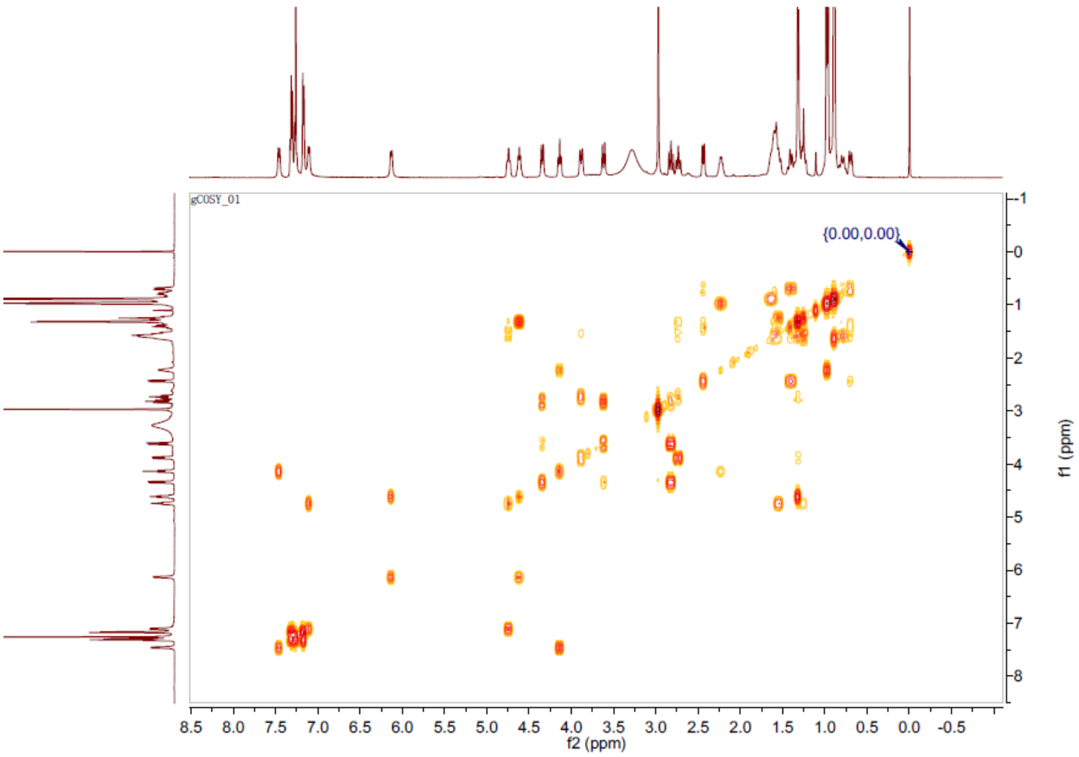

Figure S4. ^{1}H-^{1}H COSY ($CDCl_3$) spectrum of compound **1**.

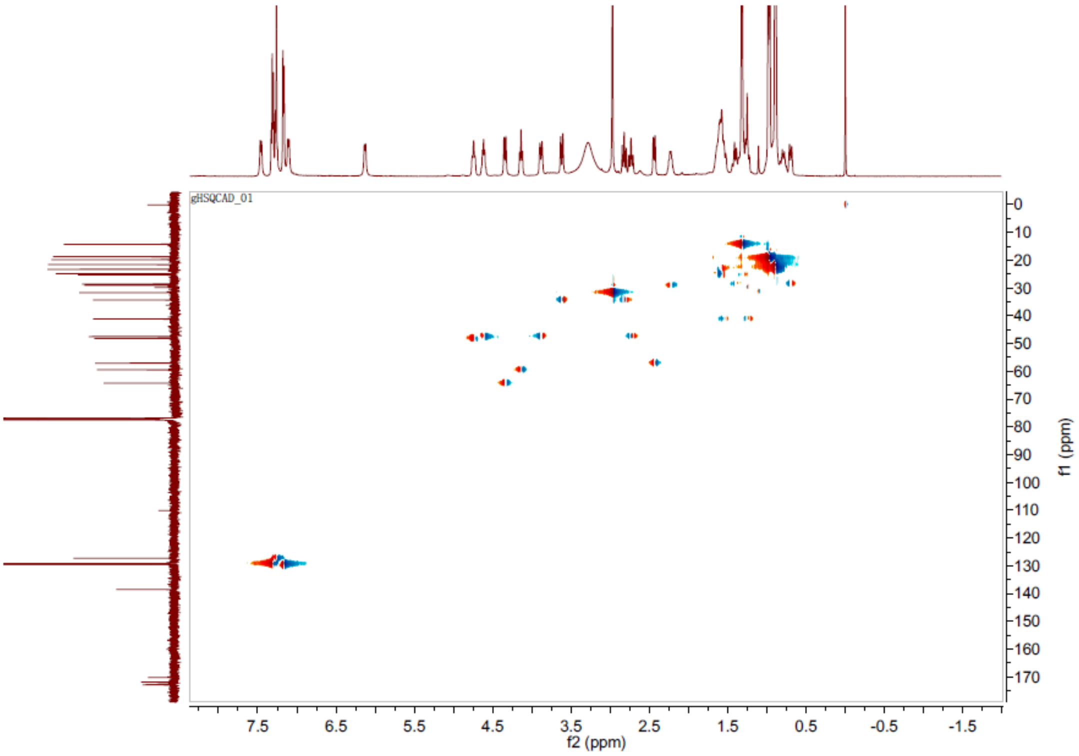

Figure S5. HSQC ($CDCl_3$) spectrum of compound **1**.

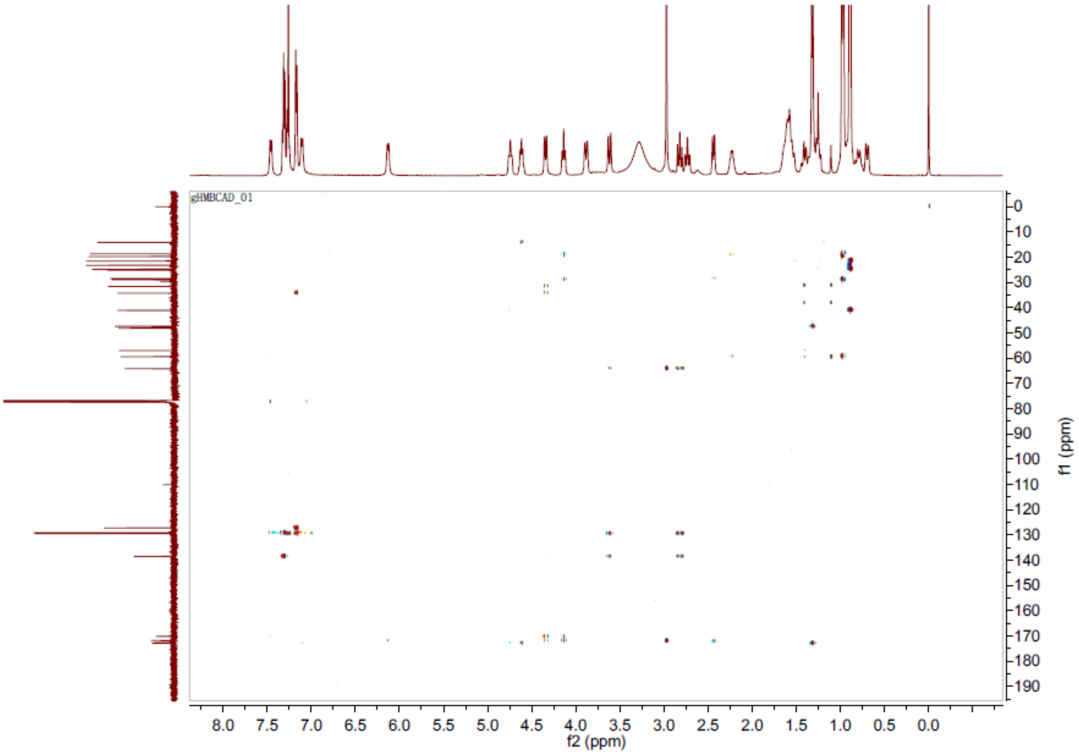

Figure S6. HMBC ($CDCl_3$) spectrum of compound **1**.

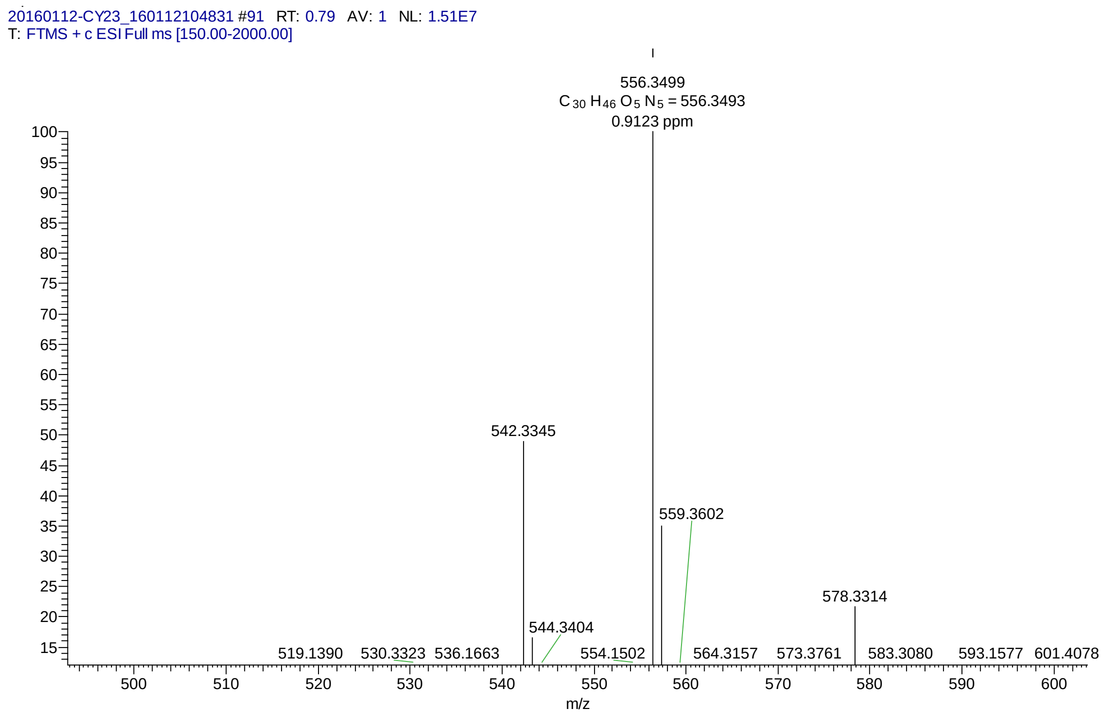

Figure S7. HRESIMS spectrum of compound **1**.

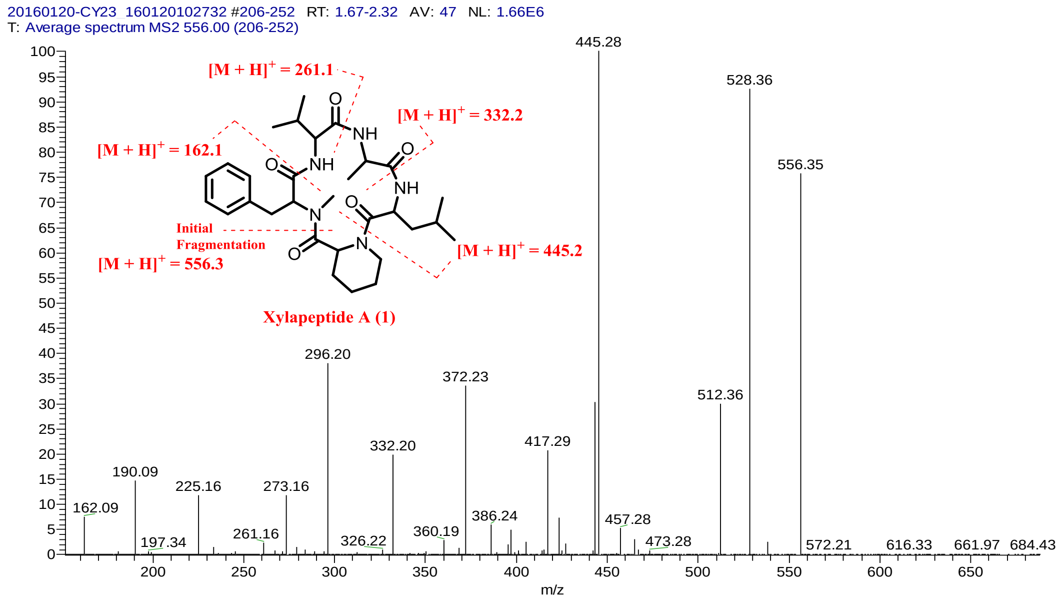

Figure S8. ESI MS[2] spectrum of compound **1**.

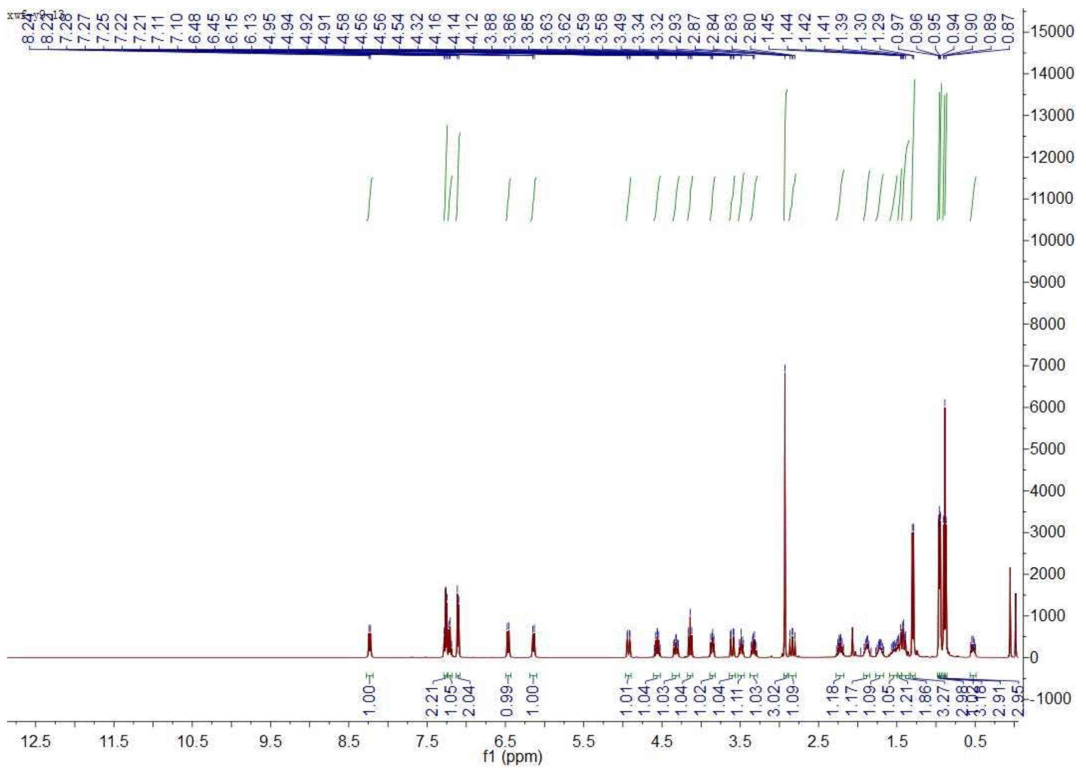

Figure S9. ^{1}H NMR (400 MHz, $CDCl_3$) spectrum of compound **2**.

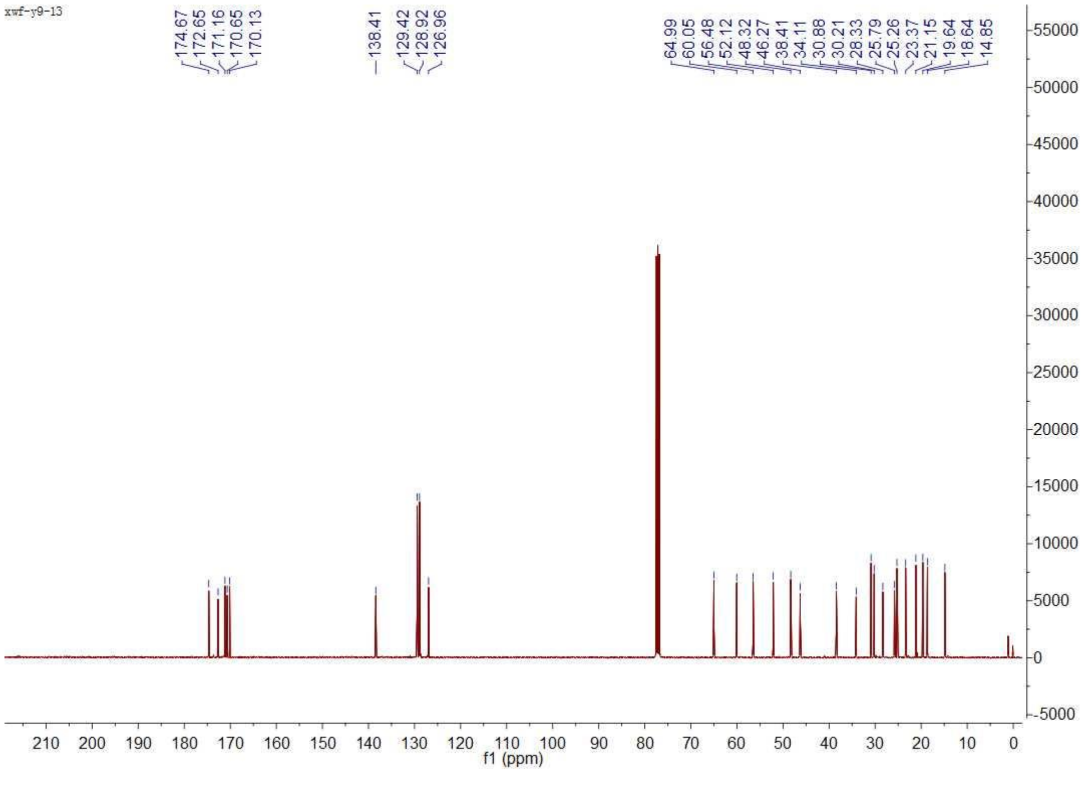

Figure S10. ^{13}C NMR (100 MHz, $CDCl_3$) spectrum of compound **2**.

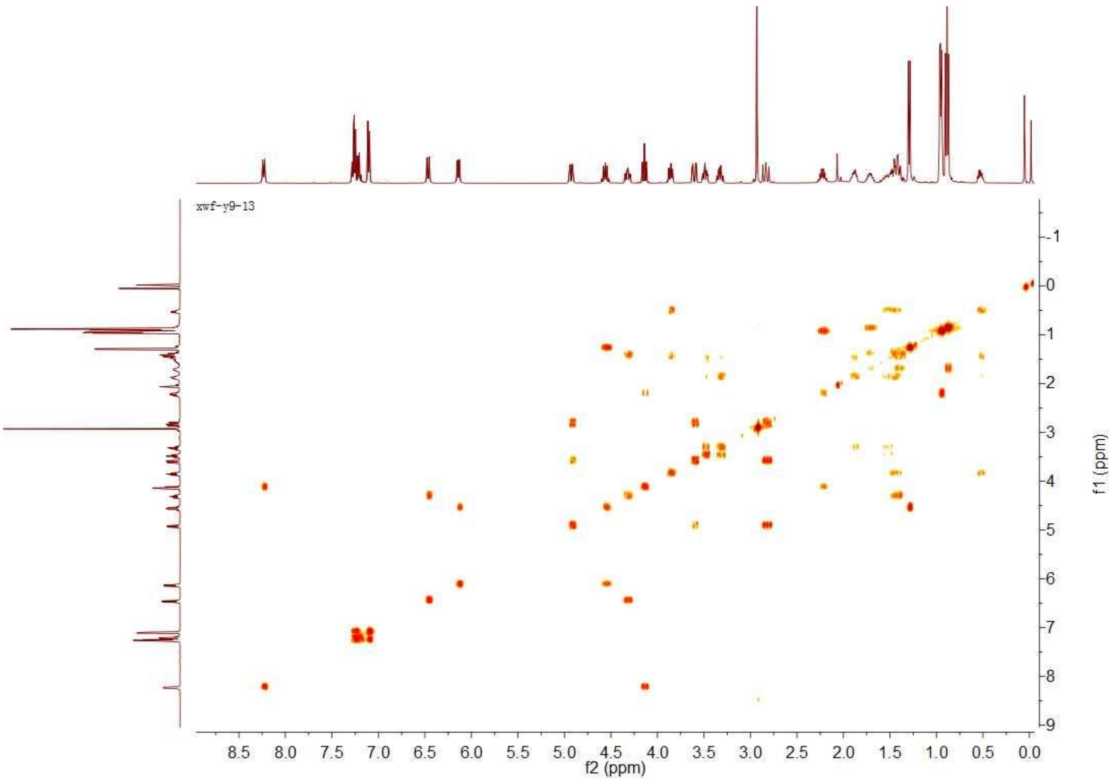

Figure S11. ^{1}H-^{1}H COSY ($CDCl_3$) spectrum of compound **2**.

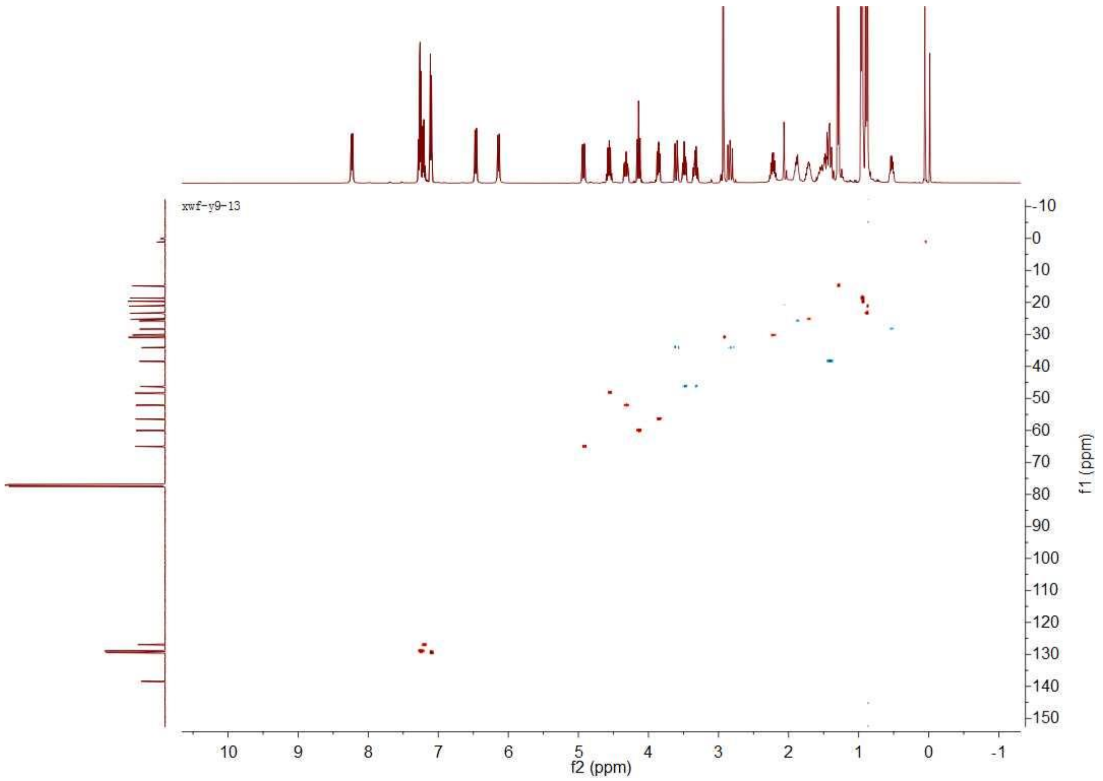

Figure S12. HSQC ($CDCl_3$) spectrum of compound **2**.

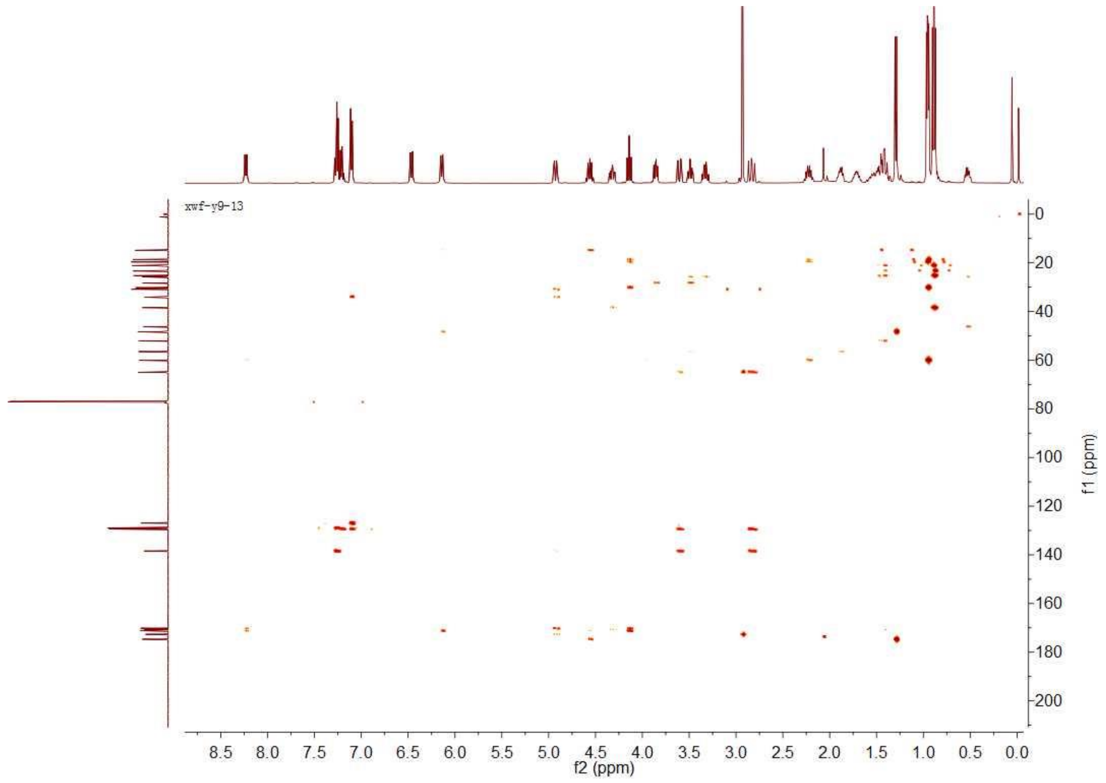

Figure S13. HMBC ($CDCl_3$) spectrum of compound **2**.

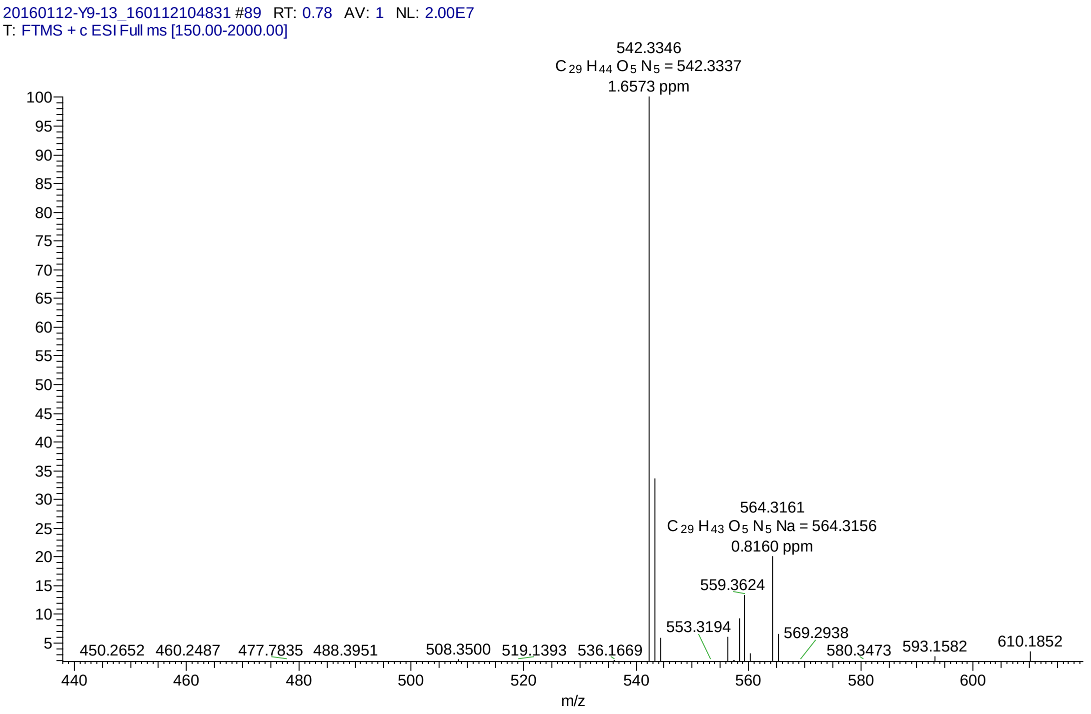

Figure S14. HRESIMS spectrum of compound **2**.

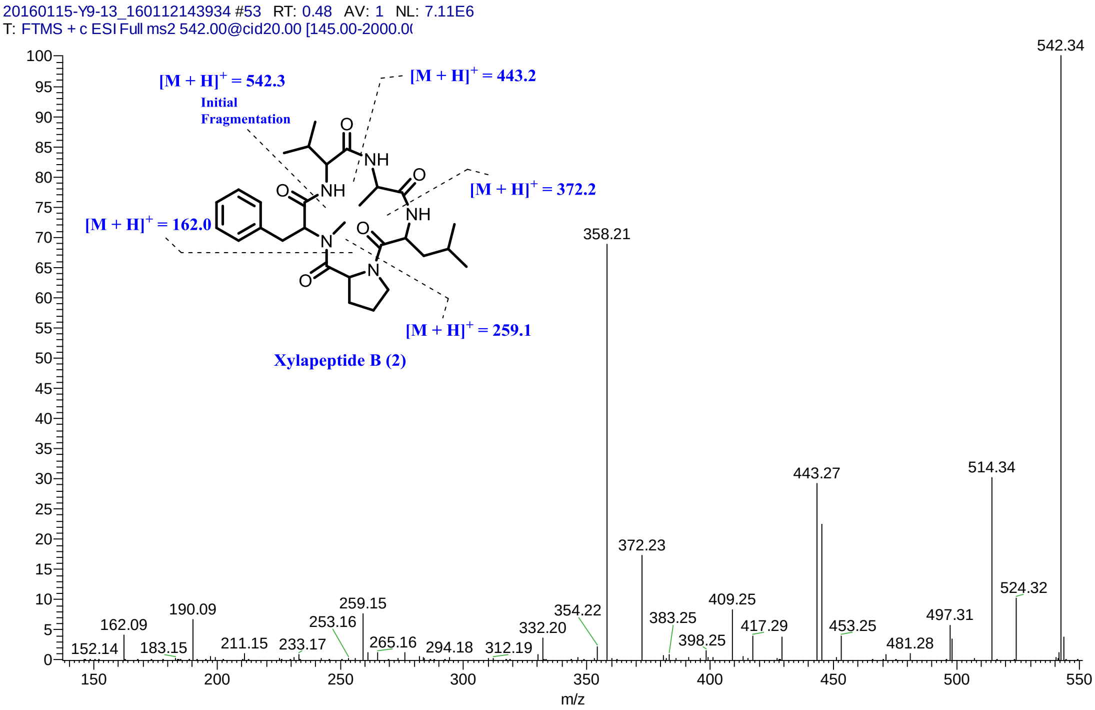

Figure S15. ESI MS^2 spectrum of compound 2.

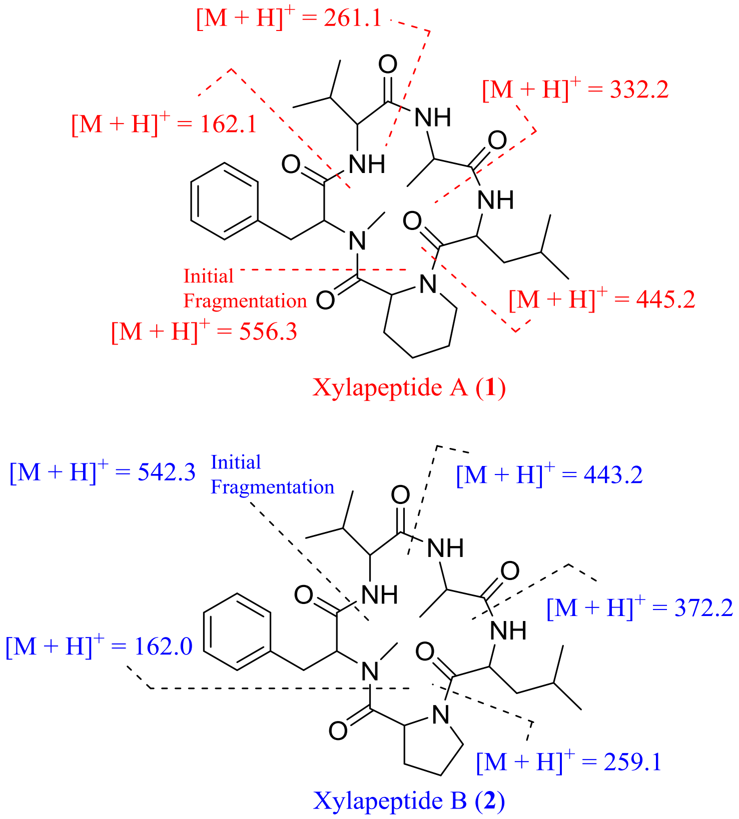

Figure S16. Assignments of ESI MS^2 fragmentation data for **1** and **2**.

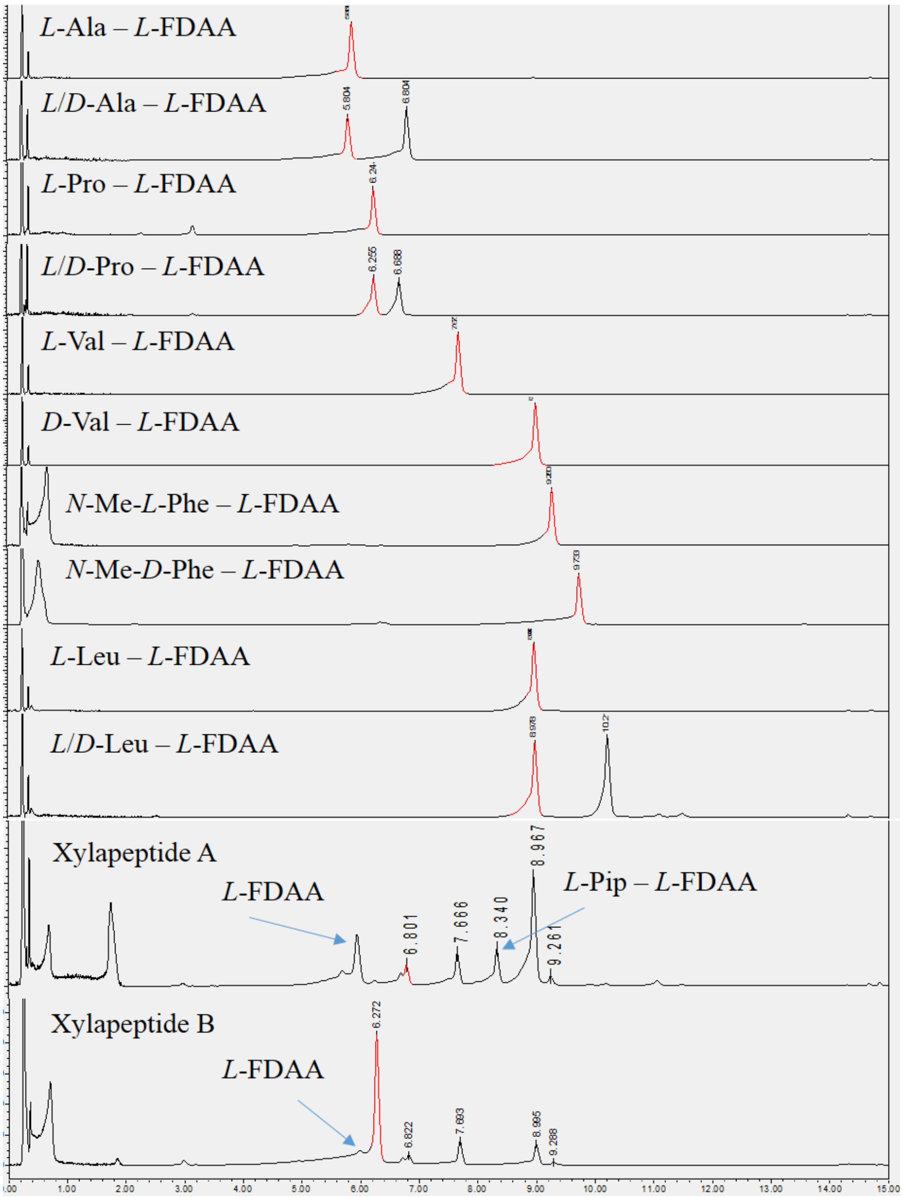

Figure S17. Marfey's analysis of the amino acids in **1** and **2** on UPLC-MS.
